# Evaluating the Mechanical Properties, and Calcium and Fluoride Release of Glass-Ionomer Cement Modified with Chicken Eggshell Powder

**DOI:** 10.3390/dj6030040

**Published:** 2018-08-18

**Authors:** Gehan Allam, Ola Abd El-Geleel

**Affiliations:** Pediatric Dentistry and Dental Public Health, Faculty of Dentistry, Ain-Shams University, Cairo 11566, Egypt; Gehan.Allam@bue.edu.eg

**Keywords:** glass-ionomer cement, eggshell powder, compressive strength, fluoride release

## Abstract

The aim of this study was to test the effect of adding chicken eggshell powder (CESP) to conventional glass-ionomer cement (GIC) on its mechanical properties, and fluoride and calcium release. CESP was added with proportions of 3% and 5% by weight to the powder component of conventional glass-ionomer cement. The specimens were categorized into group A: GIC without CESP; group B: GIC with 3% wt. CESP; and group C: GIC with 5% wt. CESP; there were 12 specimens in each group. Groups B and C showed higher compressive strength values compared to group A. However, microhardness scores were higher in group C compared to groups A and B. As for ion-release results, group B displayed the highest values of fluoride release followed by group C at both 7 and 30 days. Group C showed the highest amount of calcium release followed by both groups B and C at 7 days, while at 30 days, groups A and B showed higher calcium release compared to group C. The mechanical properties of conventional glass-ionomer restorative material were enhanced by the addition of CESP. Moreover, fluoride and calcium release were not compromised by adding CESP.

## 1. Introduction

Since its introduction in 1972, glass-ionomer cement (GIC) has been popular among clinicians due to its exclusive properties such as chemical adhesion to mineralized tissues, moisture insensitivity, and low coefficient of thermal expansion, which is close to that of tooth structures. Moreover, GIC has superior biocompatibility, fluoride release, and rechargeability, which impart its anticariogenic properties [1,2,3]. When GIC is exposed to neutral aqueous solutions after complete setting, it absorbs water and releases ions such as sodium, silica, calcium, and fluoride [4,5]. Two processes occur in relation to fluoride release: a fast burst during the early period (1–7 days), and a long-term diffusive process [6]. Despite of all these advantages, GIC has low mechanical strength properties that compromise its durability in stress-bearing areas [7]. 

Many attempts have been made to enhance the mechanical properties of conventional GIC, such as the addition of resin [8] and the incorporation of alumina, carbon, glass, hydroxyapatite, and fluoroapatite nanoparticles without compromising the fluoride-release properties of GIC [9,10].

Chicken eggshell powder (CESP) is composed of 98.2% calcium carbonate, 0.9% magnesium, and 0.9% phosphate, approximately; thus, it is considered a rich source of mineral salts, mainly calcium carbonate. Eggshell calcium is probably considered the best natural source of calcium [11]. For this particular reason, various clinical studies had been successfully conducted in the use of this rich calcium source in bone substitution [12,13], treatment of osteoporosis [14], and more recently in the remineralization of early enamel lesions [15]. 

Chicken eggshell powder (CESP) has also been used as a rich source of CaCO_3_ in order to impart mechanical reinforcement to polyethylene/polypropylene composites used in different industrial applications [16,17,18]. To date, no studies have been conducted concerning the addition of CESP with the aim of strengthening dental restorative material. The utilization of CESP as a filler material to enhance the mechanical properties of GI has some advantages, as it is naturally renewable, low-cost, and readily available. For this reason, the current study aimed to investigate the effect of adding CESP to glass-ionomer powder on its mechanical properties, as well as its fluoride and calcium release.

## 2. Materials and Methods

An anhydrous glass-ionomer restorative material (AquaCem, Dentsply, Germany) was used. This powder contains a blend of alumino-silicate glass and polyacrylic acid, which was mixed with deionized water with a ratio of 1 scoop powder: 2 drops water according to the manufacturer’s instructions.

### 2.1. Eggshell Powder Preparation

CESP was attained by calcination following the protocol of World Property intellectual organization (WO/2004/105912: Method of producing egg shell powder) [19]. This calcination process was performed to obtain pure powder free of pathogens and to increase its alkalinity. Normally, CESP contains 95% calcium carbonate, which converts to basic calcium oxide on calcination [20]. Twelve chicken eggs were cleaned with distilled water and kept in hot boiling water for 10 min at 100 °C to facilitate the removal of membranes. The egg shells were crushed and powdered to small particles with sterile mortar and pestle. The tiny, crushed particles obtained were then kept in a muffle furnace (Thermolyne 48,000) at 1200 °C to make sure the resulting powder was pathogen free. Subsequently, the size of the powder particles was measured using Mastersizer 2000.

GIC was mixed according to the manufacturer’s instructions, with 1:1 (Powder:Liquid), and CESP was added to the powder component with proportions of 3% and 5% by weight. The specimens were categorized into 3 groups:

- Group A: GIC without CESP (*n* = 12).
- Group B: GIC with 3% wt. CESP added to the powder component (*n* = 12).
- Group C: GIC with 5% wt. CESP added to the powder component (*n* = 12).

Cylindrical samples were fabricated using PTFE (Polytetrafluoroethylene) cylindrical molds with 4-mm diameter and 6-mm height for a compressive strength test [21], and other samples were fabricated with 6-mm diameter and 3-mm thickness for a surface microhardness test [22]. The molds were filled with the material, covered with PTFE tape and glass slides, flattened, and pressed in order to eliminate air bubbles from unset cement paste. A 200-g weight was placed on top of the set, thus standardizing the pressure exerted during the initial setting of the material. The samples were ejected from the molds after 30 min and stored in deionized water at 37 °C and 100% humidity for 23 h in an incubator until testing time [21]. 

### 2.2. Compressive Strength

Compressive strength was measured using a Universal testing machine (Lloyd LR 5 k, Lloyd Instruments Ltd., Hampshire, UK) with an across-head speed of 1 mm min^−1^ until failure occurred [22]. Compressive strength results were automatically calculated by dividing the maximum load before failure by the surface area using the machine operating software (Nexgen software, Version 4.6, Llyod Instruments Ltd., Hampshire, UK).

### 2.3. Microhardness Test (Vickers)

Microhardness was measured using a Vickers microhardness tester machine (Tukon1202, Wilson Hardness Tester, Instron^®^ ITW Company, Norwood, MA, USA). Three indentations were carried out for each specimen at 25 g force for 30 s, and the average score of the three readings was recorded for each specimen [23]. 

### 2.4. Fluoride and Calcium Ions Release

Twelve cylindrical specimens (6 mm in diameter and 3 mm in height) per group were prepared using mountable split Teflon rings as previously described. Equal lengths of paraffin dental floss were incorporated into the specimens during setting to suspend the specimens in the deionized water. Specimens were weighed to ensure standardization within each group using a digital balance (±0.0001 g) (Precisa 205A series, Superbal, Germany), and their dimensions (diameter and thickness) were also measured using the digital micrometer. Specimens were stored at 37 °C and 100% relative humidity for 24 h. Each specimen was then stored in an individual tightly-closed polyethylene tube containing 10 mL distilled deionized water at 37 °C. At the time of fluoride and calcium ions measurement, each specimen was removed from its container, and the storage solution was collected for analysis. The discs were then blotted dry and placed in a new container with fresh 10 mL distilled deionized water, and storage was continued [24]. Measurements of fluoride and calcium ions concentrations were made at 7 and 30 days using an ion selective electrode (Fluoride selective electrode, Orion Research, Inc., Denmark) and an atomic absorption spectrometer (Perkin-Elmer model 3100, Artisan Technology Group^®^ Shelton, Champaign, IL, USA) at 239.9 nm. Results were calculated as the amount of fluoride or calcium release per unit surface area of the specimen (μg/mm^2^).

## 3. Results

Data from all tested groups were collected, tabulated, and statistically analyzed using SPSS (21st edition, IBM corp., New York, NY, USA).

One-way ANOVA followed by Tukey’s post hoc test was used to investigate the effect of adding eggshell powder on the compressive strength of the restorative material. The results revealed that groups B (3% CESP) and C (5% CESP) showed significantly higher compressive strength values compared to group A (GI without CESP), which showed the lowest values (Table 1). 

**Figure 1:**
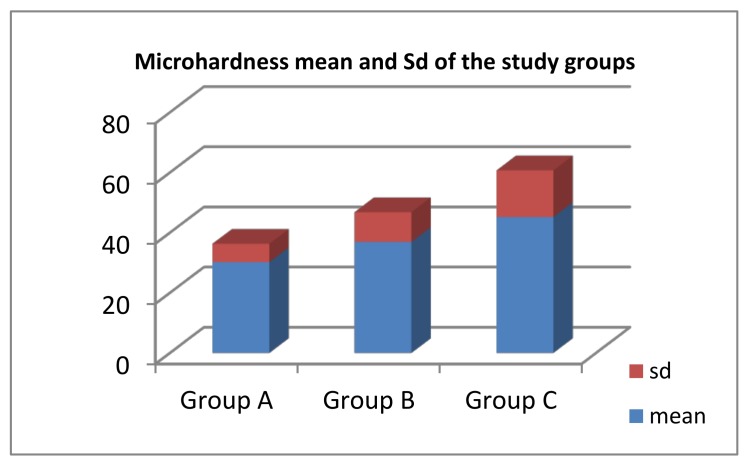
Comparison of microhardness (VHN) in relation to CESP content.

However, microhardness scores were significantly higher in group C (5% CESP) compared to groups A and B, with group A displaying the lowest microhardness values. (Figure 1)

### 3.1. Fluoride Release

One-way ANOVAs followed by Tukey’s post hoc test were used to assess the effect of adding CESP on fluoride and calcium release from the restorative material. At 7 days, group B showed the highest statistical significant value of fluoride release followed by group C, while group A showed the least significant amount of fluoride release. Similarly, at 30 days, group B showed a significantly higher amount of fluoride release compared to groups A and C, and there was no significant difference between these last two groups. An independent *t*-test of the effect of time interval on fluoride release showed that only group B had a significantly lower value of fluoride release after 30 days. Groups A and C also showed less fluoride release at 30 days than at 7 days; however, the difference was not significant for either group (Table 2).

### 3.2. Calcium Release

A one-way ANOVA followed by Tukey’s post hoc test showed that group C had a significantly higher amount of calcium release than groups A and B at 7 days, with no significant difference between these two groups. At 30 days, groups A and B showed a significantly higher amount of calcium release compared to group C.

**Figure 2:**
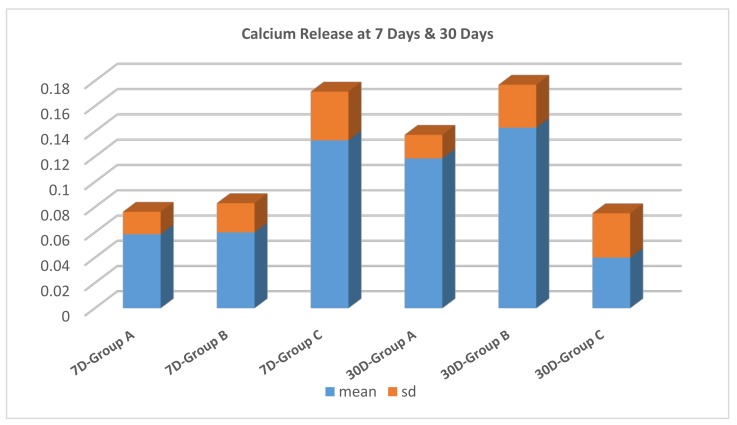
Comparisons of calcium release (mg/L) for the three groups in relation to time interval.

An independent *t*-test of the effect of time interval on calcium release showed that calcium release increased significantly at 30 days in groups A and B, while group C displayed a significantly lower amount of calcium release at 30 days, compared to values obtained at 7 days (Figure 2).

## 4. Discussion

Dental caries continues to be one of the most common childhood diseases despite the evolution in the field of oral health for children [25]. As a result, restoring decayed teeth remains one of the priorities in treatment needs. Moreover, the shorter life spans and lower biting forces of primary teeth compared to permanent teeth make all types of GIC a favorable choice to be used in children. Conventional GIC has undergone various improvements since its introduction, which in turn led to better characteristics, such as increased strength, improved handling characteristics, and enhanced wear resistance [26].

On the other hand, increasing worldwide interest in sustainable technologies led to the invention of products with lower impact on the environment [27]. Eggshell is one of the by-products of households, restaurants, and food industries, which is daily produced in massive amounts and has been categorized as one of the worst environmental problems worldwide due to its chemical composition and availability. At the same time, eggshell is considered the best natural source of calcium [28]. The utilization of eggshell in dental material industry could elevate the its added value and possibly reduce environmental pollution. Thus, the present study sought to test the effect of adding eggshell powder to the powder component of conventional GIC on some of its mechanical properties, as well as to examine whether fluoride and calcium release would be influenced by adding CESP filler in various proportions to GIC.

Compressive strength testing is the most commonly employed method to evaluate the strength of restorative materials, and surface hardness is widely used to assess the mechanical properties of restorative materials because the surface of the cement is considered to be directly affected by environmental conditions [29,30,31]. For the aforementioned reasons, we chose these two tests to evaluate the effect of adding CESP to GIC on its mechanical properties.

In the present study, it was evident that the mechanical properties of the tested specimens improved significantly with the addition of eggshell powder to conventional GIC in terms of compressive strength as well as microhardness. Furthermore, microhardness values were significantly highest in group C, followed by group B, while group A (GIC without CESP) trailed with the lowest statistically significant values in both tests. These findings reflect somehow the consistent enhancing effect of adding CESP on the mechanical properties of GIC. Since the mechanical properties of GIC improve with time, testing this novel material for extended time frames would have added extra value to the current research; however, the chosen testing time (24 h) only aimed at obtaining preliminary data to be considered as a cornerstone for future investigations.

These results are in accordance with those of Lubis et al. [32], who also concluded that eggshell filler added to acrylic resin used in the construction of denture base not only improved the mechanical properties in terms of modulus of elasticity but also provided a cost-effective and renewable filler material that could be used instead of more costly alternatives.

Similarly, Rahmi et al. [33] deduced that the incorporation of eggshell particles in cross-linked chitosan composites enhances its mechanical properties, as the values of tensile strength increased significantly with increasing proportions of added ESP; they also suggested that the utilization of this novel filler material can lead to the production of low-cost polymer composites.

The bioactivity of glass-ionomer cements stems from their capability of releasing ions, not only fluoride but also calcium and phosphates. These ions become incorporated in the nearby tooth structure to enhance its resistance to acid attacks [34]. For this reason, we were tempted to test whether adding CESP to improve the mechanical properties would affect the ion release properties of GIC.

Deionized water was selected as a medium for the estimation of fluoride and calcium ions release from the tested glass-ionomer samples; since it is devoid of any ions, it is considered a more accurate medium than artificial saliva and other acidic media [24,35,36].

Our results clearly showed that adding CESP did not interfere with the inherent ability of GIC to release ions, on the contrary, it even seemed to potentiate this ability to the extent that fluoride and calcium release were increased in groups B and C compared to GIC with no added CESP.

Kumar et al. [37] obtained similar results when they added nano-chaitosan to GIC. They found that not only did the added nanoparticles improve the mechanical properties of GIC but also the anticariogenic properties of the cement were enhanced through the recorded increase in fluoride release.

Similarly, Yli-urpo et al. [38] reported that bioactive glass (BAG) improves fluoride release in conventional GIC, which even increased with increasing proportions of BAG added to GIC. The same study reported an initial surge of the concentrations of calcium and phosphorous in the test medium followed by an evident decrease after 72 h. The authors attributed these findings to CaP precipitation, which they postulated to have precipitated around the seams of the samples and on the bottom of storage test tubes [38].

The results of the current research, however, cannot be accurately compared to the aforementioned study, since our first measurement of calcium and fluoride was done at 7 days, followed by a second measurement at 30 days; this was done for the purpose of tracing long-term release of the ions that aid in remineralization. We detected the highest values of calcium release in group C, with CESP added to GIC in a proportion of 5% by weight, followed by group B, which had a slightly lower concentration of CESP (3% wt.); this could suggest that group B had also leached out too many Ca^++^ initially (before our first measurement), before it precipitated as CaP as reported in the previous study [38], so that Ca^++^ values were depressed and came closer to the measurement obtained from group A (GI without any added CESP). At the same time, Ca^++^ in group C was not depleted by CaP precipitation, since samples in this group had a higher proportion of eggshell powder with a subsequent higher calcium content.

We can also postulate that the high calcium release in Group C (highest eggshell concentration) coupled with lower fluoride release could be explained by the ability of eggshell to absorb fluoride in solution, a theory which has been explained and utilized by various researchers [39,40] in order to find cheaper methods for the defluoridation of drinking water to avoid potential fluoride toxicity. Moreover, this could also be depicted in our results, as calcium and fluoride released in solution after 30 days were significantly lower in group C, which suggests that the “scavenging” ability of eggshell continues over time in solution to the extent of depletion of calcium and fluoride ions.

## 5. Conclusions

Within the limitations of the study, it can be concluded that:

- The mechanical properties of conventional GIC were enhanced by the addition of CESP.
- GIC unique property of fluoride release is not compromised by adding CESP to its powder component.
- Calcium release was potentiated at 5% CESP concentration, which can enhance the remineralizing ability of GIC.

## Figures and Tables

**Table 1:** Mean and standard deviation (SD) of compressive strength of three groups.

Test	A	B	C	*p*-Value
Compressive strength (MPa)	52.45 ^a^ ± 1.8	75.46 ^b^ ± 13.9	71.43 ^b^ ± 16.7	0.001

Similar superscripts letters indicate no significant difference.

**Table 2:** Mean ± standard deviation of fluoride release of the three groups in relation to time interval.

Test Interval	Group A	Group B	Group C	*p*-Value
7 days	2.03 ^aA^ ± 0.9	21.69 ^cA^ ± 0.6	4.51 ^bA^ ± 0.9	0.0001
30 days	1.33 ^aA^ ± 0.6	12.2 ^bB^ ± 4.4	3.36 ^aA^ ± 1.4	0.0001
*p*-value	0.104	0.001	0.096	

Dissimilar lowercase superscripts letters indicate significant difference in rows. Dissimilar uppercase superscripts letters indicate significant difference in columns.
